# TLR3 agonists induce fibronectin aggregation by activated astrocytes: a role of pro-inflammatory cytokines and fibronectin splice variants

**DOI:** 10.1038/s41598-019-57069-4

**Published:** 2020-01-17

**Authors:** Inge Werkman, Arend H. Sikkema, Joris B. Versluijs, Jing Qin, Pascal de Boer, Wia Baron

**Affiliations:** Department of Biomedical Sciences of Cells & Systems, section Molecular Neurobiology, University of Groningen, University Medical Center Groningen, Groningen, The Netherlands

**Keywords:** Astrocyte, Multiple sclerosis

## Abstract

Multiple sclerosis (MS) is a chronic demyelinating disease of the central nervous system which eventually results in axonal loss mainly due to failure of remyelination. Previously we have shown that the persistent presence of stable astrocyte-derived fibronectin aggregates in MS lesions impairs OPC differentiation, and thereby remyelination. Here we set out to discern whether and, if so, how inflammatory mediators as present in MS lesions trigger astrocytes to form fibronectin aggregates. Our findings revealed that in slice cultures only upon demyelination, the TLR3 agonist Poly(I:C) evoked astrocytes to form fibronectin aggregates. Consistently, pro-inflammatory cytokine-pretreated astrocytes were more susceptible to Poly(I:C)-induced fibronectin aggregation, indicating that astrocytes form fibronectin aggregates upon a double hit by inflammatory mediators. The underlying mechanism involves disrupted fibronectin fibrillogenesis at the cell surface as a result of a cytokine-induced increase in relative mRNA levels of *EIIIA*^*pos*^-*Fn* over *EIIIB*^*pos*^-*Fn* and a Poly(I:C)-mediated decrease in integrin affinity. Remarkably, fibronectin aggregation is exacerbated by white matter astrocytes compared to grey matter astrocytes, which may be a reflection of higher expression levels of EIIIA^pos^-fibronectin in white matter astrocytes. Hence, interfering with alternative fibronectin splicing and/or TLR3-mediated signaling may prevent fibronectin aggregation and overcome remyelination failure in MS lesions.

## Introduction

Multiple sclerosis (MS) is a chronic disease of the central nervous system (CNS) characterized by demyelination, inflammation and neurodegeneration in grey matter (gm) and white matter (wm)^1^. In the early stages of MS, remyelination restores axonal support and saltatory nerve conduction^2^, while upon disease progression remyelination often fails, resulting in permanent axonal loss and disease progression^1,3,4^. Studies in rodent models showed that efficient remyelination requires recruitment of oligodendrocyte progenitor cells (OPCs) into the lesion area, followed by differentiation into myelin-producing mature oligodendrocytes^3,5–7^. In parallel, an orchestrated inflammatory response is initiated to remove remyelination-inhibiting myelin debris, ultimately resulting in a local microenvironment that favors the differentiation of recruited OPCs^3,8^. Remarkably, remyelination is more efficient in gm lesions than in wm lesions^9–12^, emphasizing an important role of the local cellular and molecular environment in remyelination efficiency.

CNS demyelination is accompanied by activation of astrocytes and microglia and the release of a myriad of pro-inflammatory cytokines, immuno-regulatory factors and extracellular matrix (ECM) molecules^1,3,5,13^. ECM remodeling plays a significant role in the remyelination process. For example, the transient upregulation of fibronectin in demyelinated areas is beneficial for OPC recruitment, i.e., the early stages of remyelination, while its timely removal is required to allow OPC differentiation to proceed^14–16^. Reactive astrocytes are the primary source of the transiently deposited fibronectin upon demyelination^15,17^. In chronic demyelinated MS lesions and in lesions of an experimental model for MS, i.e., chronic relapsing experimental autoimmune encephalomyelitis (crEAE), fibronectin is not cleared and remains present in the form of stable aggregates^15^. As dimeric fibronectin^18–21^, fibronectin aggregates efficiently block myelin formation *in vitro* as well as remyelination *in vivo*^15,22^. Strikingly, while multimeric fibronectin is detected upon cuprizone-induced demyelination, fibronectin aggregates are not formed upon prolonged expression of fibronectin and demyelination^14^. Similarly, fibronectin aggregates are not observed in lysolecithin-induced demyelination experimental models and slice cultures^15,22^. Hence, fibronectin aggregates are not formed upon demyelination by default.

The fibronectin molecule contains a multitude of variable regions generated by alternative splicing of a single gene, including domains EIIIA (EDA in human) and EIIIB (EDB in human) that are specific for cellular fibronectin and which may have various functions^23,24^. For example, inclusion of either domain alters the conformation of fibronectin, thereby interfering with its integrin and cell binding properties^25–29^. Organization of fibronectin is affected by several processes, including assembly into fibronectin fibrils, proteolytic turnover, and biochemical crosslinking and restructuring. Recently, we have shown that tissue transglutaminase 2 (TG2), when exogenously supplied, can cross-link astrocyte-deposited fibronectin on a laminin substrate^30^. However, endogenously expressed TG2 does not contribute to astrocyte-mediated fibronectin aggregation, while being involved in fibronectin fibril formation^30^. In addition, proteomic analysis revealed that TG2 is not present in fibronectin aggregates^31^. Hence, the underlying mechanism of fibronectin aggregation by astrocytes is not yet known.

MS lesions contain more inflammatory mediators derived from infiltrated peripheral cells than toxin-induced lesions. For example, the pro-inflammatory cytokines IL1β, IFNγ and TNFα^32–34^ and endogenous Toll-like receptor (TLR) agonists^35–38^ are abundantly present in MS lesions. In addition, TLRs are upregulated within MS lesions and present on activated astrocytes^39^. As astrocyte activity is induced in a context-dependent manner and regulated by, among others, pro-inflammatory cytokines and TLR-mediated signaling events^39–43^, fibronectin aggregate formation may be evoked by local inflammatory mediators. Here, we aimed to clarify whether and how immune-modulating factors, known to be present in MS lesions, trigger astrocytes to produce stable fibronectin aggregates. Our findings indicate that astrocytes form fibronectin aggregates by a double hit mechanism that involves activation of astrocytes by a demyelinating event and/or exposure to pro-inflammatory cytokines via a mechanism that involves a cytokine-mediated alteration in the expression of fibronectin splice variants and a Poly(I:C)-induced decrease in fibronectin binding by astrocytes. In addition, while wm astrocytes and gm astrocytes were similar responsive to Poly(I:C), wm astrocytes formed more fibronectin aggregates than gm astrocytes *in vitro*. This may reflect the more prominent presence of fibronectin aggregates and the decreased remyelination capability in wm compared to gm MS lesions.

## Results

### TLR3 agonists induce extracellular fibronectin aggregate formation by astrocytes *in vitro*

**Figure 1:**
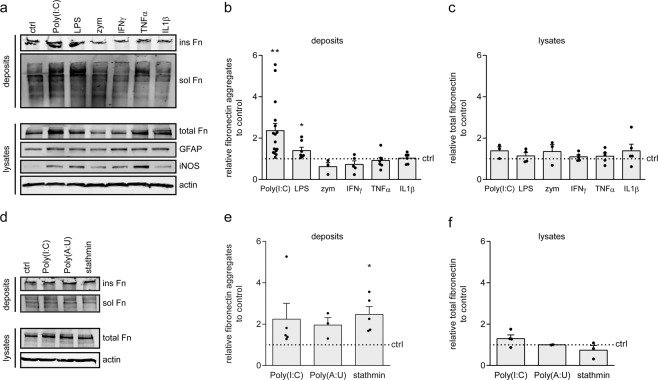
TLR3 agonists induce extracellular fibronectin aggregate formation by astrocytes. Western blot analysis of fibronectin (Fn) in DOC-soluble (sol, fibronectin dimers) and DOC-insoluble (ins, fibronectin aggregates) extracellular deposits (**a**,**b**,**d**,**e**) and Fn, GFAP and iNOS in total cell lysates (**a**,**c**,**d**,**f**) of primary rat astrocytes treated for 48 hours with pro-inflammatory cytokines IFNγ (500 units/mL), IL1β (10 ng/mL) or TNFα (10 ng/mL), or with TLR2 agonists zymosan (zym, 10 μg/mL), TLR3 agonists Poly(I:C) (50 μg/mL), Poly(A:U) (50 μg/mL), stathmin (0.5 μg/mL) and TLR4 agonist LPS (200 ng/mL). Representative blots of 3–16 independent experiments are shown in (**a**,**d)** quantification of DOC-insoluble fibronectin aggregates in deposits in **b**,**e**, and quantification of fibronectin in total cell lysates in (**c**,**f)**. Actin serves as a loading control for cell lysates; equal amounts of protein (12 μg) are subjected to DOC-(in)solubility analysis. Note that TLR3 agonists, and to a lesser extent TLR4 agonist LPS, induce fibronectin aggregation (Poly(I:C) p < 0.001; Poly(A:U) p = 0.114, ns; stathmin p = 0.018, LPS p = 0.016). Bars represent mean relative to their respective untreated control (ctrl), which was set at 1 in each independent experiment (horizontal line). Error bars show the standard error of the mean. Statistical analyses were performed using a Wilcoxon Signed Rank Test (Poly(I:C) and LPS in (**a**), failed Shapiro-Wilk normality test) or a one-sample t-test to test for differences between treatments and their respective control (*p < 0.05, **p < 0.01).

Previously, we have shown that fibronectin aggregates are formed at the relapse phase in crEAE, but not in toxin-induced models^14,15^, indicating that inflammation may contribute to fibronectin aggregation. To assess whether inflammatory mediators that are abundantly present in MS lesions contribute to fibronectin aggregation, we applied a reductionist approach and exposed primary rat astrocytes to pro-inflammatory cytokines IL1β, IFNγ and TNFα, and TLR2, TLR3, and TLR4 agonists zymosan, Poly(I:C) and LPS, respectively. Previous characterization of fibronectin aggregates indicated their insolubility in the ionic detergent sodium deoxycholate (DOC), while remaining in the stacking gel of SDS-PAGE gels^15,22,44^. Accordingly, after a 48-hour incubation with the inflammatory cytokines, extracellular deposits were extracted in DOC buffer and the extent of fibronectin aggregation was assessed by Western blot. Exposure to the TLR3 agonist and viral dsRNA mimetic Poly(I:C) significantly induced fibronectin aggregate formation (Fig. 1a,b, ins Fn, Wilcoxon Signed Rank Test p < 0.001, n = 16). Also, exposure to LPS, a bacterial membrane component that activates TLR4, reproducibly enhanced fibronectin aggregation, although to a lesser extent than Poly(I:C) (Fig. 1a,b, ins Fn, Wilcoxon Signed Rank Test p = 0.016, n = 7). In contrast, fibronectin aggregation was unchanged upon exposure to the TLR2 agonist zymosan and the pro-inflammatory cytokines IL1β, IFNγ and TNFα (Fig. 1a,b, ins Fn). Total cellular fibronectin expression was similar at all conditions (Fig. 1a,c, lysates). For all treatments, GFAP expression was increased compared to untreated astrocytes (Fig. 1a), indicating that astrocytes were activated. In addition, expression of iNOS, known to be differentially upregulated in astrocytes in response to inflammatory mediators^45^, was uncorrelated with the extent of fibronectin aggregation (Fig. 1a). Thus, Poly(I:C) induced-fibronectin aggregation is not a reflection of enhanced astrocyte activation. Exposure to the endogenous TLR3-agonist protein stathmin also increased the amount of DOC-insoluble fibronectin aggregates in deposits compared to untreated astrocytes (Fig. 1d,e, stathmin p = 0.017, n = 5), while no significant alterations in overall fibronectin expression were detected (Fig. 1d,f). The more selective TLR3 agonist Poly(A:U) also reproducibly, but not significantly, increased aggregation of fibronectin (Fig. 1d,e, Poly(A:U) p = 0.114, ns, n = 3). Hence, our findings indicate that TLR3 agonists, and to a lesser extent a TLR4 agonist, induced extracellular fibronectin aggregate formation by astrocytes.

### TLR3 agonist Poly(I:C) decreases astrocyte adhesion to fibronectin

**Figure 2:**
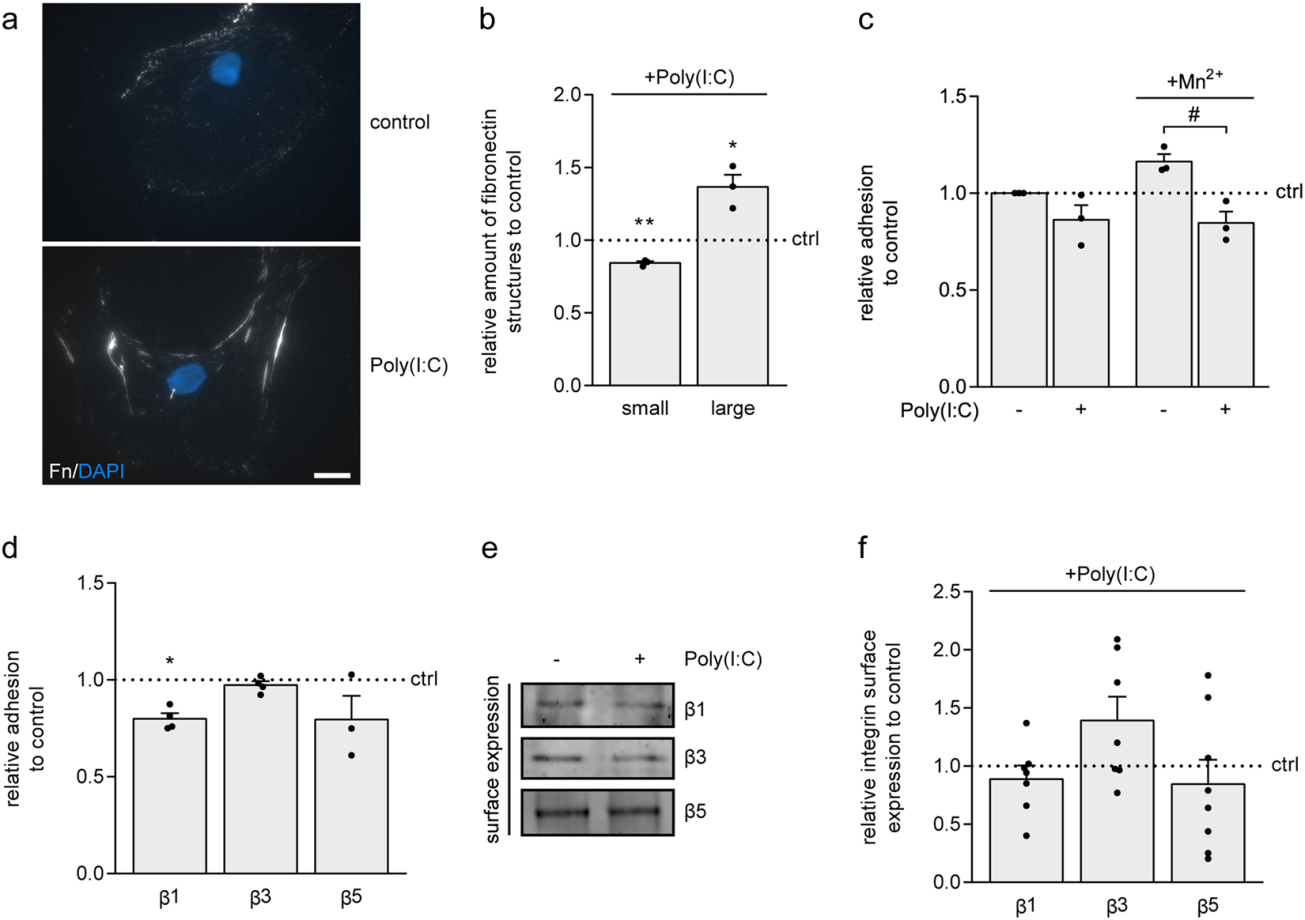
Poly(I:C) induces formation of large elongated-structures of fibronectin at the cell surface, and decreases cell adhesion to fibronectin. (**a**,**b**) Fibronectin (Fn) immunocytochemistry of living primary rat astrocytes treated with Poly(I:C) (50 μg/mL) for 48 hours. Representative images of 4 independent experiments are shown in (**a)** quantification of cells with scattered small fibronectin structures and cells with large elongated fibronectin structures at the cell surface in (**b**). Note that Poly(I:C) decreases the amount of scattered fibronectin (p = 0.006) and increases the amount of fibril-like fibronectin (p = 0.048) at the cell surface. (**c**) Adhesion assay of astrocytes to fibronectin (n = 3). Primary rat astrocytes were left untreated or treated with Poly(I:C) (50 μg/mL) for 2 hours, in the absence or presence of Mn^2+^ (1 mM), known to increase integrin affinity. Note that Poly(I:C) decreases astrocyte adhesion to fibronectin even in the presence of Mn^2+^ (p = 0.011). (**d**) Adhesion assay of astrocytes to fibronectin in the absence or presence of functional blocking antibodies against integrin β1, β3 or β5 (n = 3–4). Note that astrocytes primarily bind to fibronectin via integrin β1. (**e**,**f**) Western blot analysis of cell surface expression of integrin β1, β3 and β5 of primary rat astrocytes treated with Poly(I:C) (50 μg/mL) for 48 hours. lysates; equal amounts of protein (100 μg) are subjected to immunoprecipitation. Representative blot of 7–8 independent experiments are shown in (**e**), quantification in (**f**). Bars represent means relative to their respective untreated control (ctrl), which was set at 1 for each independent experiment (horizontal line). Error bars show the standard error of the mean. Statistical analyses were performed using a one-sample t-test to test for differences between treatments and their respective controls (*p < 0.05, **p < 0.01). An unpaired t-test was used to test for differences in adhesion between untreated and Poly(I:C)-treated cells in the absence or presence of Mn^2+^ (^#^p < 0.05). Scale bar is 20 µm.

In most cell types, fibronectin is assembled into a fibrillar matrix. This process initiates at the cell surface primarily via binding to integrin α5β1, and is supported by binding to integrin αvβ3^23,46–50^. Upon exposure to Poly(I:C), i.e., at fibronectin aggregating conditions, fibronectin assembled at the cell surface of astrocytes in a clustered manner, forming large, elongated structures (Fig. 2a). In contrast, untreated astrocytes showed a more diffuse staining and smaller, punctuated structures of fibronectin at the cell surface (Fig. 2a,b, small p = 0.006, n = 3; large p = 0.048, n = 3). Moreover, Poly(I:C) reduced the adhesion of astrocytes to fibronectin in the presence of Mn^2+^, a known enhancer of integrin affinity^51^ (Fig. 2c, Poly(I:C) + Mn^2+^ *versus* Mn^2+^ p = 0.011, n = 3). This indicates that Poly(I:C) interfered with binding of fibronectin to integrins, its main receptors at the cell surface. An adhesion assay in the presence of integrin-specific blocking antibodies showed that astrocytes primarily adhered to fibronectin via integrin β1 (Fig. 2d, β1 p = 0.006, n = 4). Selective blocking of integrin β3 and integrin β5 did not significantly affect adhesion of astrocytes to fibronectin (Fig. 2d). Cell surface biotinylation of untreated and Poly(I:C)-treated astrocytes, followed by immunoprecipitation of integrin β1, β3 or β5 and visualization of surface integrins by Western blotting and streptavidin detection revealed that surface expression of integrin β1, β3 and β5 was virtually similar in untreated and Poly(I:C)-treated cells (Fig. 2e,f). Hence, Poly(I:C) interfered not with the cell surface levels of integrin β1, β3 and β5, but rather modulated integrin affinity that may result in more localized and large fibril-like structures at the cell surface.

### White matter astrocytes generate more fibronectin aggregates than grey matter astrocytes

**Figure 3:**
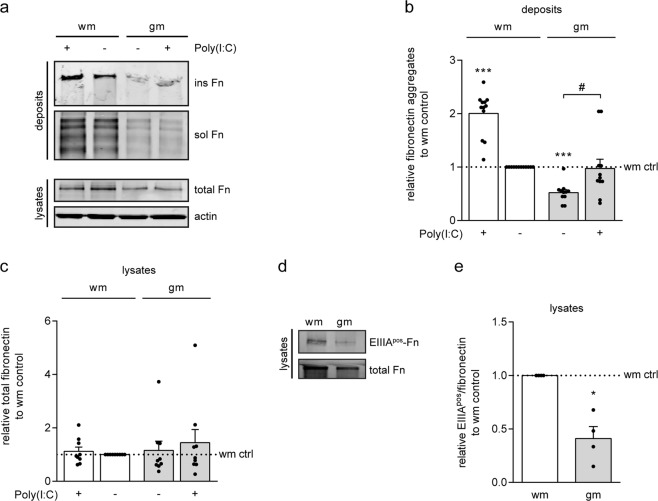
White matter astrocytes generate more fibronectin aggregates than grey matter astrocytes. (**a**–**c**) Western blot analysis of fibronectin (Fn) in DOC-soluble (sol, fibronectin dimers) and DOC-insoluble (ins, fibronectin aggregates) extracellular deposits (**a**,**b**) and total cell lysates (**a**,**c**) of primary rat grey matter (gm) and white matter (wm) astrocytes treated with Poly(I:C) (50 μg/mL) for 48 hours. Representative blots of 11–12 independent experiments are shown in (**a**), quantification of DOC-insoluble fibronectin aggregates in deposits in (**b**) and quantification of fibronectin in total cell lysates in (**c**). Actin serves as a loading control for total cell lysates; equal amounts of protein (12 μg) are subjected to DOC-(in)solubility analysis. Note that wm astrocytes generate a higher absolute amount of fibronectin aggregates than gm astrocytes (p = 0.001). (**d**,**e**) Western blot analysis of EIIIA^pos^-fibronectin in total cell lysates of gm and wm astrocytes. Representative blots of 4 independent experiments are shown in (**d**), quantification of EIIIA^pos^-fibronectin of total fibronectin is shown in **e**. Note that wm astrocytes express more EIIIA^pos^-fibronectin than gm astrocytes (p = 0.013). Bars represent mean relative to wm astrocyte control (ctrl), which was set at 1 for each independent experiment (horizontal line). Error bars show the standard error of the mean. Statistical analysis was performed using a one-sample t-test (*p < 0.05, ***p < 0.001) to test for differences between Poly(I:C) treated wm astrocytes and wm control astrocytes (wm ctrl), a Wilcoxon Signed Rank Test was used to test for differences with wm control, and an Mann Whitney test was used to test for differences between untreated and Poly(I:C)-treated gm astrocytes (wm + Poly(I:C), gm ctrl and gm + Poly(I:C) failed Shapiro-Wilk normality test) (^#^p < 0.05).

Fibronectin aggregates have been examined in wm MS lesions^15^, while the above experiments were performed with cortical, i.e., gm-derived, astrocytes. Given the reported functional differences between gm and wm astrocytes^52–54^, we next examined whether wm astrocytes also form fibronectin aggregates in response to TLR3 agonist Poly(I:C). Fibronectin aggregation was enhanced by approximately 2-fold upon Poly(I:C) exposure in both wm and gm astrocytes (Fig. 3a,b, wm + Poly(I:C), Wilcoxon Signed Rank Test p < 0.001, n = 12; gm + Poly(I:C) *versus* gm untreated, Mann Whitney test p = 0.012, n = 11), while cellular fibronectin levels were comparable (Fig. 3a,c). Intriguingly, wm astrocytes generated twice as much fibronectin aggregates than their gm counterparts (Fig. 3a,b, Wilcoxon Signed Rank Test p = 0.001, n = 11). As the cellular EIIIA- and EIIIB-fibronectin splice variants have been implicated in fibronectin fibril formation at the cell surface^25–29^, we next examined the expression levels of these splice variants in gm and wm astrocytes. Western blot analysis revealed that *in vitro* gm astrocytes expressed a lower proportion of EIIIA^pos^-fibronectin than wm astrocytes (Fig. 3d,e gm p = 0.013, n = 4). Unfortunately, no suitable antibody to detect fibronectin that contains EIIIB is available. Hence, these findings showed that wm astrocytes generated more fibronectin aggregates than gm astrocytes, which may relate to elevated levels of EIIIA^pos^-fibronectin in wm astrocytes, while the susceptibility of either astrocyte to Poly(I:C)-induced fibronectin aggregation was similar.

### Poly(I:C) induces fibronectin aggregation only after demyelination in *ex vivo* organotypic cerebellar slice cultures

**Figure 4:**
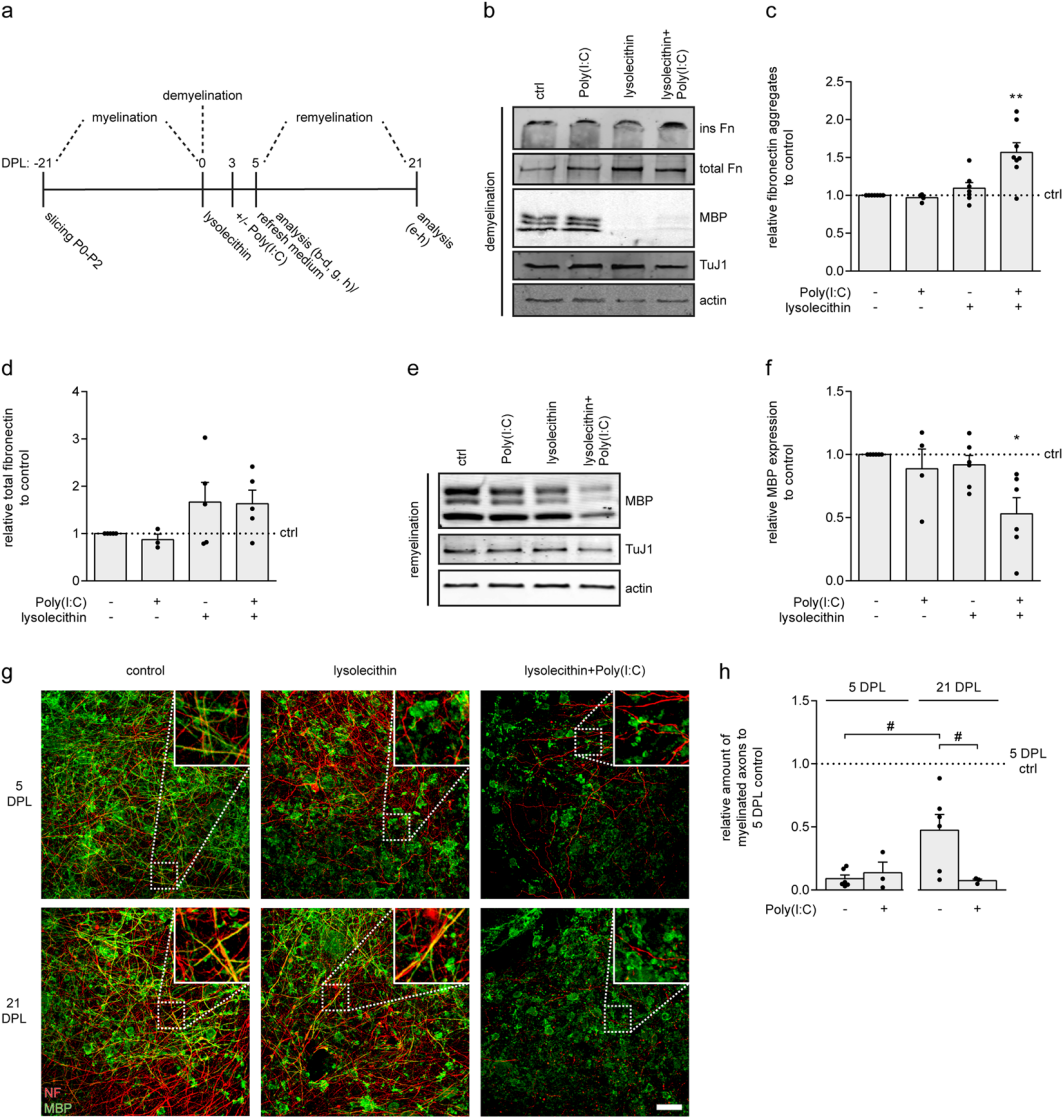
TLR3 agonist Poly(I:C) induces fibronectin aggregation in organotypic cerebellar slice cultures only after demyelination, and inhibits remyelination. (**a**) Schematic representation of treatments and analysis. Organotypic cerebellar slice cultures obtained from newborn rats were cultured for 3 weeks to allow for myelination. At day 0, demyelination was induced by lysolecithin. At 2 days post lysolecithin (DPL), demyelinated slices were left untreated or treated with the TLR3 agonist Poly(I:C) (50 μg/mL) for 48 hours after which slices were washed and new medium was added. Remyelination was allowed until 21 DPL. (**b**–**f**) Western blot analysis of fibronectin (Fn), MBP (myelin marker) and TuJ1 (neuronal marker) in demyelinated slices 5 DPL (**b-d**) and remyelinated slices 21 DPL. (**e**,**f**) Representative blots of 3–8 independent experiments are shown in (**b**,**e**) and quantification in (**c**,**d**,**f**). Actin was used as a loading control. (**g**,**h**) Immunohistochemistry for myelin (MBP, green) and axons (neurofilament, NF-H, red) at 5 and 21 DPL. Representative images of 3–6 independent experiments are shown in (**g**), quantification of the % myelinated axons in (**h**) relative to 5 DPL control. Bars represent mean relative to untreated myelinated control (ctrl), which was set at 1 for each independent experiment (horizontal line). Error bars show the standard error of the mean. Statistical analyses were performed using a one-sample t-test to test for differences with control slices (*p < 0.05, **p < 0.01), and a one way ANOVA with a Šidák multiple comparisons post-test was used to test for differences in remyelination on 21 DPL between lysolecithin and lysolecithin + Poly(I:C)-treated slices (# p < 0.05). Scale bar is 50 µm.

To more closely mimic demyelination conditions, we next examined whether TLR3 agonist Poly(I:C) also induces fibronectin aggregation in *ex vivo* organotypic cerebellar slice cultures. To prevent that Poly(I:C) interferes with the initial events of demyelination, myelinated organotypic cerebellar slice cultures were first subjected to lysolecithin-induced demyelination and allowed to recover for 2 days. The demyelinated slice cultures were subsequently exposed to Poly(I:C) for another 2 days (Fig. 4a). Western blot analysis demonstrated that sequential treatment with lysolecithin and Poly(I:C) significantly increased fibronectin aggregation compared to control and lysolecithin-treated cerebellar slice cultures (Fig. 4b,c, lysolecithin + Poly(I:C) p = 0.003, n = 8). Poly(I:C) treatment of myelinated cultures, thus without prior demyelination, did not result in increased fibronectin aggregation (Fig. 4b,c). Total levels of fibronectin were not significantly altered (Fig. 4b,d). Notably, while the expression of a major myelin-specific protein myelin basic protein (MBP) was decreased upon lysolecithin treatment, MBP expression levels in Poly(I:C)-treated myelinated cerebellar slice cultures were similar to MBP levels in untreated myelinated cerebellar slice cultures (Fig. 4b). This indicates that transient exposure to Poly(I:C) alone did not induce demyelination. Expression of neuron-specific class III beta-tubulin, a neuronal marker recognized by the TuJ1 antibody, was unaffected in all conditions (Fig. 4b). In this slice culture model, spontaneous remyelination is observed approximately three weeks after lysolecithin-induced demyelination^22,24,55^. To assess whether Poly(I:C)-induced fibronectin aggregation interfered with OPC maturation, as observed upon intralesional injection of fibronectin aggregates in toxin-induced demyelinated lesions *in vivo*^15,22^, the extent of OPC maturation was determined at 21 days post lysolecithin (DPL)-induced demyelination (Fig. 4a). Western blot analysis revealed that MBP levels in lysolecithin-treated cerebellar slice cultures almost returned to the levels of untreated slice cultures (Fig. 4e,f). In contrast, MBP expression in lysolecithin and Poly(I:C)-treated cerebellar slice cultures remained significantly decreased (Fig. 4e,f, lysolecithin + Poly(I:C) p = 0.014, n = 6). Exposure of myelinated slice cultures to Poly(I:C) had no effect on MBP expression levels after 21 days (Fig. 4e,f). Double labelling of MBP with the axonal marker neurofilament (NF-H) revealed that addition of Poly(I:C) to lysolecithin-demyelinated cerebellar slice cultures significantly decreased the percentage of myelinated axons at 21 DPL compared to the percentage of myelinated axons in lysolecithin-treated slices (Fig. 4g,h, lysolecithin + Poly(I:C) n = 3, *versus* lysolecithin n = 6, p = 0.047). Thus, exposure to Poly(I:C) induced fibronectin aggregation only after demyelination, which resulted in impaired MBP re-expression and remyelination. As these findings showed that in slice cultures only activated astrocytes were susceptible to Poly(I:C)-induced fibronectin aggregate formation, we next examined whether pre-incubation with pro-inflammatory cytokines makes astrocytes more prone to Poly(I:C)-induced fibronectin aggregation.

### Pre-incubation with pro-inflammatory cytokine potentiates Poly(I:C)-induced fibronectin aggregation

**Figure 5:**
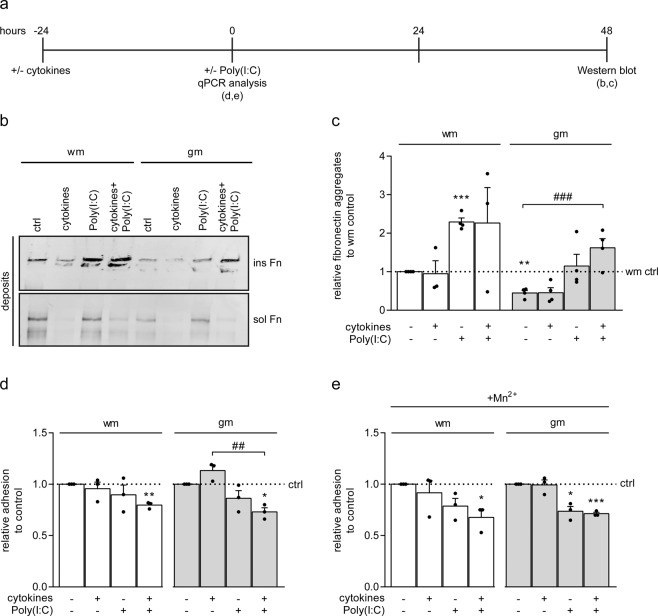
Pre-incubation with pro-inflammatory cytokines potentiates Poly(I:C)-induced fibronectin aggregation by grey matter astrocytes. (**a**) Schematic representation of astrocyte treatment and analysis. Primary rat grey matter (gm) and white matter (wm) astrocytes are pre-treated for 24 hours with a mixture of IFNγ (500 units/mL), IL1β (10 ng/mL) and TNFα (10 ng/mL), followed by Poly(I:C) treatment (50 μg/mL) for 48 hours. (**b**,**c**) Western blot analysis of fibronectin (Fn) in DOC-soluble (sol, fibronectin dimers) and DOC-insoluble (ins, fibronectin aggregates) extracellular deposits. Representative blots of 3–4 independent experiments are shown in (**b**), quantification of DOC-insoluble aggregated fibronectin in **c**. Equal amounts of protein (12 μg) are subjected to DOC-(in)solubility analysis. Note that pre-incubation with cytokines potentiates fibronectin aggregation by gm (p = 0.004), but not wm astrocytes. (**d**,**e**) Adhesion assay of astrocytes to fibronectin (n = 3). Astrocytes were treated for 1 hour with a mixture of IFNγ (500 units/mL), IL1β (10 ng/mL) and TNFα (10 ng/mL), followed by adhesion for 2 hours in the absence or presence of Poly(I:C) (50 μg/mL) and/or the absence (**d**) or presence (**e**) of Mn^2+^ (1 mM), which is known to increase integrin affinity. Note that pre-incubation with cytokines potentiates the decreased effect of Poly(I:C) on adhesion (wm + cytokines + Poly(I:C) p = 0.008; gm + cytokines + Poly(I:C) p = 0.022), which cannot be overcome by Mn^2+^ (wm + cytokines + Poly(I:C) p = 0.048; gm + cytokines + Poly(I:C) p = 0.002). Bars represent mean relative to wm astrocyte control (wm ctrl, **c**) or untreated control (ctrl, **d**,**e**), which was set at 1 for each independent experiment (horizontal line). Error bars show the standard error of the mean. Statistical analyses were performed using a one-sample t-test to test for differences with wm astrocyte control (ctrl, **c**) or their respective untreated control (**d**,**e**) (*p < 0.05, **p < 0.01, ***p < 0.001). A one way ANOVA with a Dunnett multiple comparison post-test was used to compare treatment groups with untreated gm control (**c**) (^###^p < 0.001) and a one way ANOVA with a Šidák multiple comparisons post-test was used to test for differences between treatment groups (**d**,**e**) (^##^p < 0.01).

To assess a putative role of cytokines in fibronectin aggregation, gm and wm astrocytes were pre-exposed to a mixture of pro-inflammatory cytokines IFNγ, IL1β and TNFα for 24 hours, followed by stimulation with Poly(I:C) for 48 hours (Fig. 5a). Pre-incubation of gm astrocytes with pro-inflammatory cytokines increased the level of fibronectin aggregates generated upon Poly(I:C) treatment compared to untreated astrocytes (Fig. 5b,c, gm-control *versus* gm + cytokines + Poly(I:C) p = 0.005, n = 4). However, the amount of fibronectin aggregates generated upon cytokines + Poly(I:C) treatment by gm astrocytes were still considerably less than of Poly(I:C)-treated wm astrocytes that were not pre-exposed to cytokines (Fig. 5b,c). The extent of Poly(I:C)-induced fibronectin aggregation was similar for cytokine-treated and untreated wm astrocytes (Fig. 5b,c). Fibronectin aggregation was not enhanced upon treatment with a mixture of pro-inflammatory cytokines alone in either type of astrocyte (Fig. 5b,c), which is in line with exposure to the single cytokines (Fig. 1a,b). Notably, cytokine treatment appeared to decrease the total amount of deposited fibronectin (Fig. 5b). As Poly(I:C) treatment reduced the binding of gm astrocytes to fibronectin (Fig. 2), next was examined whether cytokines affect cell adhesion. Exposure to pro-inflammatory cytokines alone did not affect adhesion of gm and wm astrocytes to fibronectin, however, pre-incubation with cytokines followed by Poly(I:C) treatment resulted in a significant decrease in astrocyte adhesion to fibronectin of both wm and gm astrocytes (Fig. 5d wm + cytokines + Poly(I:C) p = 0.008; gm + cytokines + Poly(I:C) p = 0.022, n = 3), which was more prominent in the presence of Mn^2+^ (Fig. 5g, wm + cytokines + Poly(I:C) p = 0.048; gm + cytokines + Poly(I:C) p = 0.002, n = 3). Thus, pre-incubation with pro-inflammatory cytokines may enhance fibronectin aggregation by potentiating the negative effect of Poly(I:C) on astrocyte adhesion to fibronectin.

### Pro-inflammatory cytokines enhance the *EIIIA*^*pos*^- to *EIIIB*^*pos*^*-Fn* mRNA ratio

**Figure 6:**
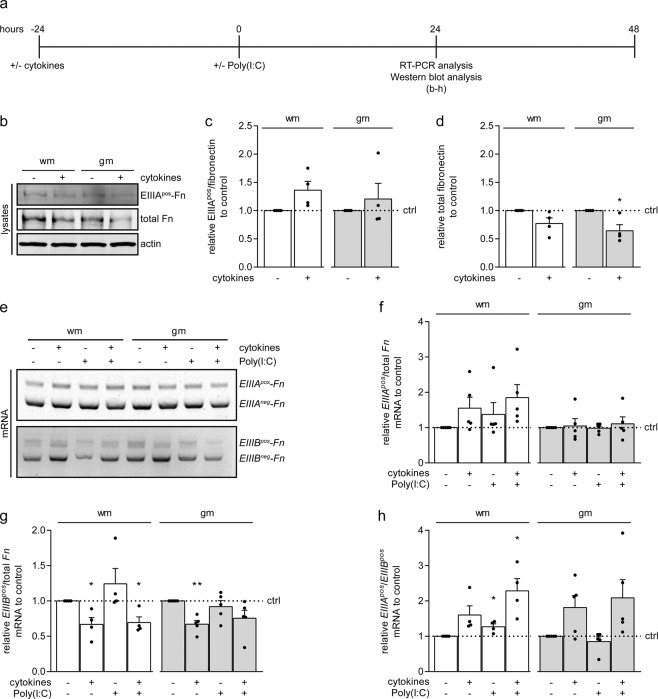
Pro-inflammatory cytokines favor *EIIIA*^*pos*^ over *EIIIB*^*pos*^ splicing in primary rat astrocytes. (**a**) Schematic representation of astrocyte treatment and analysis. Primary rat grey matter (gm) and white matter (wm) astrocytes were pre-incubated for 24 hours with a mixture of IFNγ (500 units/mL), IL1β (10 ng/mL) and TNFα (10 ng/mL), and left untreated or treated with Poly(I:C) (50 μg/mL) for 24 hours. (**b**–**d**) Western blot analysis of EIIIA^pos^-fibronectin and total fibronectin in total cell lysates. Representative blots of 4 independent experiments are shown in (**b**), quantification of EIIIA^pos^-fibronectin of total fibronectin in (**c**) and total fibronectin in (**d**). Actin serves as a loading control. (**e**–**h**) RT-PCR analysis of *EIIIA*^*pos*^-, *EIIIA*^*neg*^-, *EIIIB*^*pos*^- and *EIIIB*^*neg*^-*Fn* mRNA. Representative agarose gels of 4–5 independent experiments are shown in **e**, quantification of *EIIIA*^*pos*^/total *Fn*, *EIIIB*^*pos*^/total *Fn*, and *EIIIA*^*pos*^/*EIIIB*^*pos*^-*Fn* mRNA ratios in (**f**,**g**,**h)** respectively. Note that *EIIIB*^*pos*^-*Fn* mRNA, but not *EIIIA*^*pos*^-*Fn* mRNA levels are decreased in cytokine pre-treated astrocytes (wm + cytokines p = 0.043; wm + cytokines + Poly(I:C) p = 0.031; gm + cytokines p = 0.003; gm + cytokines + Poly(I:C) p = 0.094, ns), resulting in an enhanced *EIIIA*^*pos*^/*EIIIB*^*pos*^-*Fn* mRNA ratio (wm + cytokines p = 0.104, ns; wm + cytokines + Poly(I:C) p = 0.044; gm + cytokines p = 0.075, ns; gm + cytokines + Poly(I:C) p = 0.102, ns). Bars represent mean relative to their respective control astrocytes (ctrl), which was set at 1 for each independent experiment (horizontal line). Error bars show the standard error of the mean. Statistical analyses were performed using a one-sample t-test to test for differences with their respective control astrocytes (*p < 0.05, **p < 0.01).

As an increased level of EIIIA^pos^-fibronectin in wm astrocytes correlated with increased fibronectin aggregation (Fig. 3a,b,d,e), and EIIIA^pos^-fibronectin is involved in cell adhesion^25,26,28,29^, the effect of pro-inflammatory cytokines on EIIIA^pos^-fibronectin levels was examined next. Astrocytes were treated for 24 hours with pro-inflammatory cytokines, and cultured for another 24 hours (Fig. 6a). Exposure to pro-inflammatory cytokines reproducibly, but not significantly, increased the proportion of EIIIA^pos^-fibronectin in wm astrocytes, but not in gm astrocytes (Fig. 6b,c, wm + cytokines p = 0.100, ns, n = 4), while total fibronectin levels substantially decreased upon cytokine treatment in wm astrocytes (Fig. 6b,d, wm + cytokines p = 0.125, ns, n = 4) and significantly decreased in gm astrocytes (Fig. 6b,d, gm + cytokines p = 0.046, n = 4). As to the best of our knowledge, an antibody for EIIIB that is suitable for Western blotting is not available, the mRNA expression of alternatively spliced *EIIIA*^*pos*^*-Fn* and *EIIIB*^*pos*^-*Fn* was examined. To this end, a semi-quantitative RT-PCR using primers that span each of the alternatively spliced *Fn* exons was performed. Using this method, primer pairs produce two different products, i.e., a larger product when the alternatively spliced exon was included and a smaller product if it was spliced out (Fig. 6e). When normalized to total *Fn* mRNA levels, i.e., *EIIIA*^*pos*^-*Fn* + *EIIIA*^*neg*^-*Fn*, a considerable increase in *EIIIA*^*pos*^-*Fn* mRNA levels was observed upon exposure to cytokines, alone or in combination with Poly(I:C) treatment in wm astrocytes (Fig. 6e,f, wm + cytokines p = 0.148, ns; wm + cytokines + Poly(I:C) p = 0.082, ns, n = 5), which is consistent with the substantial increase in protein expression shown by Western blot analysis (Fig. 6b,c). In contrast, the mRNA levels of *EIIIB*^*pos*^-*Fn* decreased in both wm and gm astrocytes upon cytokine pre-incubation and remained decreased following stimulation with Poly(I:C) (Fig. 6e,g, wm + cytokines p = 0.043, n = 4; wm + cytokines + Poly(I:C) p = 0.031, n = 4; gm + cytokines p = 0.003, n = 5; gm + cytokines + Poly(I:C) p = 0.094, ns, n = 5). This resulted in a significantly enhanced *EIIIA*^*pos*^/*EIIIB*^*pos*^-*Fn* mRNA ratio in Poly(I:C)-stimulated cytokine-pretreated wm astrocytes, while the ratio was reproducibly increased in cytokine-treated wm astrocytes (Fig. 6e,h, wm + cytokines p = 0.104, ns, n = 4; wm + cytokines + Poly(I:C) p = 0.034, n = 4). A similar trend was observed with gm astrocytes (Fig. 6e,h, gm + cytokines p = 0.075, ns, n = 5, gm + cytokines + Poly(I:C) p = 0.102, ns, n = 5). The *EIIIA*^*pos*^/*EIIIB*^*pos*^-*Fn* mRNA ratio moderately, but significantly, increased upon Poly(I:C) treatment alone in wm astrocytes (Fig. 6e,h, wm + Poly(I:C) p = 0.044, n = 4), which in contrast to pre-incubation with cytokines was not a result of decreased *EIIIB*^*pos*^-*Fn*. Thus, upon pre-incubation with cytokines of gm and wm astrocytes, and upon Poly(I:C) treatment of wm astrocytes, the relative mRNA levels of *EIIIA*^*pos*^-*Fn* compared to the *EIIIB*^*pos*^-*Fn* mRNA levels were increased. As the cellular fibronectin molecule contains either the EIIIA or the EIIIB domain, or both combined, this indicates there is a relative increase of fibronectin containing solely the EIIIA domain. To visualize fibronectin splice variants at the astrocyte surface and/or in extracellular aggregates, non-permeabilized cells were co-labelled with an antibody that recognizes fibronectin that lacks EIIIB and an antibody that recognizes total fibronectin. Upon pre-incubation with cytokines and subsequent exposure to Poly(I:C), thus at conditions where fibronectin aggregates were generated (Fig. 5b,c), large extracellular structures located in between cells showed clear immunoreactivity to both EIIIB^neg^-fibronectin and total fibronectin (Suppl. Fig. S1a,b, arrows), while EIIIB^neg^-fibronectin was hardly visible at the cell surface. Similar extracellular structures that were enriched for EIIIB^neg^-fibronectin were visible upon exposure to Poly(I:C) alone, but to a lesser extent than Poly(I:C)-treated cytokine-pre-incubated astrocytes. Thus, the extracellular fibronectin aggregates that were not attached to the cell surface likely contained fibronectin that lacks the EIIIB domain and/or may have underwent a conformational change that increased the accessibility for the anti-EIIIB^neg^-fibronectin antibody.

### Enhanced *EDA*^*pos*^*-FN* to *EDB*^*pos*^*-FN* mRNA ratio in cultured MS astrocyte**s**

**Figure 7:**
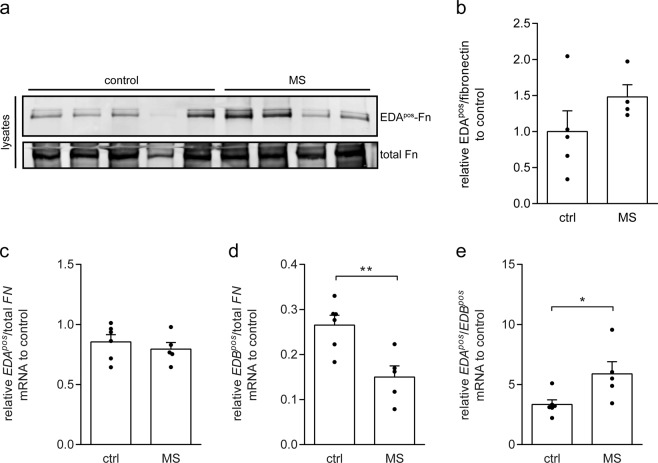
Increased *EDA*^*pos*^-*FN* to *EDB*^*pos*^-*FN* mRNA ratio in MS astrocytes. Western blot analysis of total fibronectin and EDA^pos^-fibronectin in total cell lysates (**a**,**b**) of control (5 healthy donors) and MS (4 MS donors) astrocytes cultures. Blot is shown in (**a**), quantification of EDA^pos^-fibronectin of total fibronectin in (**b**). (**c**–**e)** qPCR analysis of *EDA*^*pos*^/total *FN* (**c**), *EDB*^*pos*^/total *FN* (**d**), and *EDA*^*pos*^/*EDB*^*pos*^-*FN* mRNA (**e**) ratios in control (6 healthy donors) and MS (5 MS donors) astrocyte cultures. Note that the *EIIIB*^*pos*^-*FN*; mRNA levels of total *FN* mRNA levels are decreased in MS astrocytes (p = 0.0046), resulting in an enhanced *EIIIA*^*pos*^-*FN* to *EIIIB*^*pos*^-*FN* mRNA ratio (p = 0.015). Bars represent means (**c**–**e**) or means relative to the average ratio of astrocytes obtained from postmortem tissue of healthy subjects, which was set at 1 (**b**). Error bars show the standard error of the mean. Statistical analyses were performed with a two-sided t-test to test for differences between control and MS astrocytes (**b**–**e**) (*p < 0.05, **p < 0.01).

Our previous findings demonstrated that cultured wm astrocytes obtained from MS patients (MS astrocytes), but not wm astrocytes obtained from healthy subjects (control astrocytes), possessed the ability to form fibronectin aggregates without prior exposure to cytokines and in the absence of Poly(I:C) treatment^15^. Given that an enhanced presence of EIIIA^pos^ fibronectin over EIIIB^pos^ fibronectin correlated with fibronectin aggregation, the EDA^pos^-fibronectin protein levels and *EDA*^*pos*^/*EDB*^*pos*^*-FN* mRNA ratio in MS astrocytes were examined next. Western blot analysis revealed that with the exception of astrocytes obtained from one healthy subject, the proportion of EDA^pos^-fibronectin of total fibronectin in MS astrocytes was substantially increased compared to control astrocytes (Fig. 7a,b, ctrl n = 5 *versus* MS p = 0.212, ns, n = 4). In addition, qPCR analysis showed that while the *EDA*^*pos*^-*FN* mRNA levels over total *Fn* were virtually similar (Fig. 7c), *EDB*^*pos*^-*FN* mRNA levels were significantly decreased in MS astrocytes compared to control astrocytes (Fig. 7d, MS *versus* control p = 0.005, n = 6). As a consequence, the *EDA*^*pos*^/*EDB*^*pos*^-*FN* mRNA ratio was higher in MS astrocytes than in control astrocytes (Fig. 7e, MS n = 5 *versus* control p = 0.015, n = 6). Thus, similar to pro-inflammatory cytokine-treated rat astrocytes, the relative *EDB*^*neg*^-*FN* mRNA levels were decreased in MS astrocytes compared to control astrocytes, indicating that as in primary rat astrocytes, an increase in *EIIIA*^*pos*^/*EIIIB*^*pos*^-*FN* mRNA ratio correlated with enhanced fibronectin aggregation.

## Discussion

Persistent ECM deposition in demyelinated MS lesions impedes remyelination^15,16,56^. This includes reactive astrocytes that form fibronectin aggregates which persist in MS lesions and impair OPC maturation^15^. Our present findings indicate that sequential activation of primary astrocytes by respectively a mixture of pro-inflammatory cytokines and TLR3 agonist Poly(I:C), increased fibronectin aggregation. Similarly, in organotypic cerebellar slice cultures, Poly(I:C) only induced fibronectin aggregation upon demyelination. Poly(I:C) treatment induced fibronectin aggregation to a similar extent by both primary gm and wm astrocytes, while wm astrocytes formed a higher absolute amount of fibronectin aggregates than gm astrocytes. Pro-inflammatory cytokines increased the relative mRNA levels of the alternatively spliced *EIIIA*^*pos*^-*Fn* over *EIIIB*^*pos*^-*Fn*, which, together with subsequent Poly(I:C) exposure, decreased the binding of fibronectin to the surface of astrocytes. Consistently, fibronectin aggregate-forming MS astrocytes had a higher *EDA*^*pos*^/*EDB*^*pos*^-Fn mRNA ratio than control astrocytes. Hence, both *in vitro* and *ex vivo*, fibronectin aggregates are formed by a double hit, i.e., first a pro-inflammatory cytokine-mediated activation of astrocytes followed by exposure to TLR3 agonists, which *in vivo* includes protein agonist stathmin^57^. This respectively interferes with alternative fibronectin splicing and fibronectin cell surface binding, making astrocytes more prone to form fibronectin aggregates.

While fibronectin aggregates are not formed upon lysolecithin-induced demyelination *in vivo*^15,22^ and in cerebellar slice cultures, Poly(I:C) treatment of demyelinated, but not myelinated, cerebellar slice cultures, induced fibronectin aggregation. Likely, in toxin-induced demyelinated lesions the activated astrocytes may not encounter a TLR3 agonist during the transient period of fibronectin deposition, and therefore do not form fibronectin aggregates. In MS lesions, the prolonged presence of fibronectin as a consequence of a decreased ability to clear fibronectin by the lack of fibronectin (aggregate) clearing matrix metalloproteinases, such as MMP7^44^, may increase the chance of encountering a TLR3 agonist as a double hit. In addition, due to inefficient removal of myelin debris, activated astrocytes may encounter endogenous TLR3 protein agonist stathmin, an otherwise intracellular myelin protein. Stathmin indeed induced fibronectin aggregate formation *in vitro*, is abundantly present in MS lesions, and mostly found in inflamed areas^36,57^. Moreover, TLR3 expression on the surface of astrocytes is enhanced^39^, and co-localizes with stathmin in MS lesions^36,39,57^. Notably, TLR3 recognizes dsRNA, and although some viruses are implicated in MS pathogenesis, these are mainly ssRNA viruses^58^, and thus it is not likely that these viruses are involved in fibronectin aggregation. Therefore, increased accessibility of the endogenous TLR3 protein agonist stathmin in myelin debris combined with the prolonged presence of fibronectin, increases the change of a double hit, likely resulting in fibronectin aggregation in MS lesions. It remains however to be determined whether other cells in the cerebellar slice cultures may have contributed to fibronectin aggregation and/or remyelination failure, as microglia and oligodendrocytes also recognize Poly(I:C) via TLR3^59,60^. Of relevance in this respect, Poly(I:C) did not induce fibronectin aggregation and/or demyelination in myelinated slice cultures.

**Figure 8:**
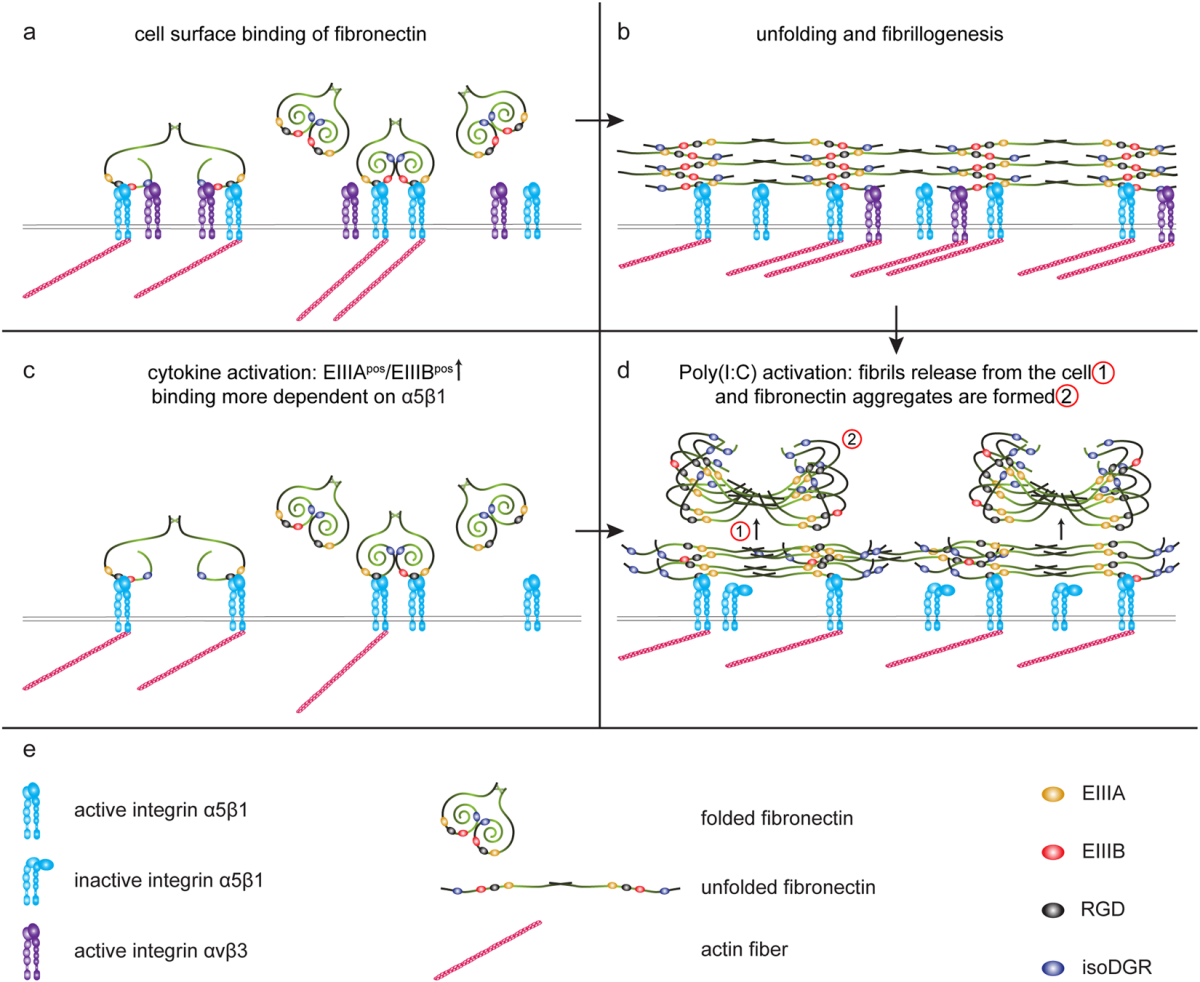
Proposed model of fibronectin aggregation by astrocytes. (**a**,**b**) Schematic representation of fibronectin fibrillogenesis^46–49,62,67^. Under normal circumstances, fibronectin molecules bind primarily at the astrocyte surface to integrin α5β1, and to a lesser extent integrin αvβ3 (**a**). Fibronectin binding to the astrocyte surface unfolds fibronectin that allows self-association of fibronectin molecules to from a fibril matrix (**b**). (**c**,**d**) Schematic representation of fibronectin aggregation. Upon exposure to a pro-inflammatory environment, as present in MS lesions, relatively more EIIIA/EDA^pos^-fibronectin than EIIIB/EDB^pos^-fibronectin is produced. (**c**) This results in enhanced binding to integrin α5β1, reduced binding to integrin αvβ3, and impartial unfolding of fibronectin. (**c**) Upon a second-hit with a TLR3 agonist, the binding of fibronectin to the astrocyte surface is reduced, resulting in detachment (1) and refolding (2) of the fibronectin multimers in aggregates. (**d**) Notably, when EIIIA/EDA^pos^-fibronectin levels are already high, fibronectin aggregates may be formed without prior exposure to pro-inflammatory cytokines (**b**,**d**). (**e**) Description of the used elements and symbols.

Our present findings indicate that dysregulated fibronectin fibrillogenesis may underlie fibronectin aggregation. In a cell-dependent manner, deposited fibronectin can be assembled into a branched, fibrillar network or matrix that plays an active role in providing environmental information to cells that encounter it, among others, by functioning as a scaffold for other bioactive signaling proteins^61,62^. Fibronectin matrix assembly is a complex multistep process, initiated upon binding of soluble dimeric fibronectin to activated cell surface integrin α5β1 (Fig. 8a) followed by unfolding, self-association of different fibronectin dimers and other ECM proteins into linear multimeric fibrils (Fig. 8b)^46–48,50,63^. Integrin αvβ3 also contributes to fibronectin fibrillogenesis^23,49,64^. Importantly, the binding strength of integrins to fibronectin is an essential part for the initiation and propagation of fibril formation. Both alternatively spliced domains EIIIA and EIIIB, although not essential, have been implicated in the level and stability of fibronectin fibril formation. More specifically, inclusion of the EIIIA domain enhances binding to integrin receptor α5β1^65^, whereas EIIIB inclusion enhances cell adhesion to fibronectin via binding of integrin αvβ3^66,67^. Thus, under normal circumstances, fibronectin binds to astrocytes via integrin α5β1, and likely also via integrin αvβ3, which supports the formation of fibronectin fibrils (Fig. 8a,b). Our data demonstrated that in pro-inflammatory cytokine-activated astrocytes, the relative *EIIIA*^*pos*^-*Fn* over *EIIIB*^*pos*^-*Fn* mRNA levels were increased, which may strengthen the binding of fibronectin to integrin α5β1, and reduce binding to integrin αvβ3 (Fig. 8c). In addition, fibronectin without EIIIB is less able to make fibrils^27^, resulting in impartial refolding (Fig. 8c,d). Integrins transmit signals bi-directionally, i.e., via outside-in and inside-out signaling^68,69^. The observed inability of Mn^2+^ to increase the integrin affinity of Poly(I:C)-treated astrocytes may be caused by a loss of inside-out activation of integrins upon exposure to Poly(I:C). This is consistent with a previous study that reports that Poly(I:C) decreases cell adhesion to fibronectin by a loss of inside-out signaling of active integrin β1^70^. Thus, exposure to TLR3 agonist Poly(I:C) reduced the binding of astrocytes to fibronectin (Fig. 8d). Remarkably, pre-incubation with cytokines potentiated the Poly(I:C)-mediated loss of cell adhesion to fibronectin, likely as fibronectin binding to the surface of cytokine-activated astrocytes rely more on integrin α5β1, due to a decrease in EIIIB^pos^-fibronectin^27,65^. In Poly(I:C)-treated cytokine-pre-incubated astrocytes, non-cell associated fibronectin aggregates were visualized by an antibody that recognizes fibronectin that lacks EIIIB, while EIIIB^neg^-fibronectin was not recognized at the cell surface. Hence, as a consequence of Poly(I:C)-mediated loss of integrin affinity, the formed fibronectin fibrils may be released from the surface (Fig. 8d,1), refold and form aggregates (Figs. 8d, 2). Indeed, unfolded non-cell associated fibronectin is prone to form aggregates^71^. Additionally, inclusion of EIIIB makes fibronectin more sensitive to proteolysis^72^, indicating that a slower degradation and prolonged presence of fibronectin due to the lack of EIIIB may increase the change of a double hit activation-induced aggregate formation. Also, EIIIB^neg^-fibronectin in aggregates are more stable and less vulnerable to extracellular proteases^72^. Whether the detached fibronectin fibrils or multimers may act as seeds for further self-association of fibronectin and other ECM molecules and ultimately form aggregates remains to be determined.

Astrocytes adopt distinct phenotypes in response to their environment, and it should be taken into account that astrocyte responses to inflammatory mediators are regionally heterogeneous^52,73^. We show that Poly(I:C) treatment induced a 2-fold increase in fibronectin aggregation by both cultured gm and wm astrocytes, while also exposure to cytokines increased the *EIIIA*^*pos*^/*EIIIB*^*pos*^-*Fn* mRNA in both gm and wm astrocytes. However, while the response to inflammatory mediators appeared similar, wm astrocytes formed a higher absolute amount of fibronectin aggregates than gm astrocytes. In fact, Poly(I:C)-treated wm astrocytes do not require a pre-incubation with cytokines to produce a maximum amount of aggregates *in vitro*. This may be partly due to the higher relative endogenous EIIIA^pos^-fibronectin levels in wm astrocytes than in gm astrocytes, and that the maximum amount of fibronectin aggregates had been reached. Also, Poly(I:C) treatment without cytokine pre-incubation modestly favored *EIIIA*^*pos*^-*Fn* over *EIIIB*^*pos*^-*Fn* splicing by wm astrocytes but not gm astrocytes. To what extent differences in regional fibronectin aggregation are responsible for the differences in remyelination capability in gm and wm MS lesions remains to be clarified. Cortical gm lesions occur without significant infiltration of haematogenous leukocytes and show less astrocyte reactivity compared to wm MS lesions^74,75^. This is primarily visible in type 1 leukocortical lesions. In these gm and wm spanning lesions, the gm area shows greater remyelination efficiency, which may be a reflection of lower astrocyte reactivity, higher number of cells of the oligodendrocyte lineage, regional heterogeneity of OPCs, and decreased intralesional ECM deposition^10,12,76^. The presence of fibronectin aggregates in gm MS lesions remains to be determined, but based on the less inflammatory character of gm lesions^75^, the absence of fibronectin immunoreactivity in marmoset gm EAE lesions^77^ and our present findings, it is tempting to hypothesize that in gm MS lesions less remyelination-impairing fibronectin aggregates are formed than in (chronic) wm MS lesions.

Remarkably, postmortem-derived MS wm astrocytes form fibronectin aggregates in the absence of cytokines and TLR3 agonists^15^. MS astrocytes have been exposed to the pathological environment and have encountered pro-inflammatory cytokines that may have altered their fibronectin splicing as well as TLR3 agonists, which may explain their ability to form fibronectin aggregates *in vitro* in the absence of TLR3 agonists. Alternatively, an inherent difference in alternative fibronectin splicing between control and MS astrocytes, as for example observed for cultured gm and wm astrocytes, cannot be excluded. Following this reasoning, it would be interesting to examine whether control and MS gm astrocytes differ in alternative fibronectin splicing and aggregate formation. Intriguingly, as in cytokine-activated primary rat astrocytes, the *EDA*^*pos*^/*EDB*^*pos*^-*FN* mRNA ratio was higher in MS astrocytes than in control astrocytes, as a consequence of reduced *EDB*^*pos*^-*FN* mRNA levels. Upon toxin-induced demyelination, astrocytes are also exposed to pro-inflammatory cytokines, while fibronectin aggregates are not detected *in vivo*^14,15,22^. Our previous findings revealed that *FN* mRNA levels, including *EIIIA*^*pos*^- and *EIIIB*^*pos*^-*FN* transcripts, are increased in lysolecithin-induced demyelinated lesions at 5 DPL compared to unlesioned control^15,16^. Additional analysis of these data revealed that the mRNA ratio of *EIIIA*^*pos*^/*EIIIB*^*pos*^-*FN* rather decreased than increased upon lysolecithin-induced demyelination compared to unlesioned controls at 5 DPL (0.52 ± 0.02, p = 0.01, n = 4), which is still evident at 14 DPL (0.57 ± 0.04, p = 0.046, n = 3), i.e., at remyelinating conditions. This may explain why astrocytes in lysolecithin-induced lesions are less susceptible to from fibronectin aggregates.

Taken together, we propose a double-hit model for fibronectin aggregation involving an initial activation in response to pro-inflammatory cytokines (first hit), which interferes with alternative fibronectin splicing, followed by a response to TLR3 agonists (second hit) that decreases integrin affinity (Fig. 8). Regional heterogeneity is reflected by the increased amounts of fibronectin aggregates formed by wm astrocytes compared to gm astrocytes. Preventing fibronectin aggregate formation may prove beneficial as an approach for remyelination enhancing-based treatment of MS. Thus, factors that interfere with alternative fibronectin splicing and TLR3-mediated signaling, and/or factors that prevent the decreased fibronectin-integrin binding aid to proper fibril formation, thereby precluding aggregation, are potential targets to aid efficient remyelination in MS. As remyelination following demyelination is essential for axonal survival and restoration of saltatory conduction^1–4^, restoring remyelination in MS may be an effective treatment in halting disease progression and reversing disability.

## Methods

### Cell culture

#### Rat astrocytes

Animal protocols were approved by the Institutional Animal Care and Use Committee of the University of Groningen. All methods were carried out in accordance with national and local experimental animal guidelines and regulations. Primary gm rat astrocytes of either sex were isolated from the neonatal cortex and wm astrocytes from neonatal non-cortical parts, which mainly consist of wm, by a shake off procedure and cultured as described^19,59,76^. Astrocytes were cultured at a density of 1 × 10^6^ cells/10 cm-dish, 50,000 cells/13-mm poly-L-lysine (5 µg/ml)-coated coverslip, or 20,000 cells/8-well Permanox chamber slide. When indicated, one hour after plating, cells were subjected to the inflammatory mediators IFNγ (500 units/mL), TNFα (10 ng/mL), IL1β (10 ng/mL), or TLR2 agonist zymosan (10 μg/mL), TLR3 agonist polyinosinic:polycytidylic acid (Poly(I:C), 50 μg/mL), TLR3 agonist polyadenylic-polyuridylic acid (Poly(A:U), 50 μg/mL), TLR3 agonist stathmin (0.5 μg/mL), or TLR4 agonist lipopolysaccharide (LPS, 200 ng/mL) for 48 hours.

#### Human astrocytes

Autopsy samples of human white matter brain material were obtained from the Netherlands Brain Bank. All donors from whom material was collected had signed written informed consent for brain autopsy and the use of material and clinical information for research purposes. All methods were carried out in accordance with relevant guidelines and regulations. Control donors (5 female/1 male) were of ages between 63 and 94, and mean postmortem delay was 7.2 hours. MS donors (4 female/2 male) were of ages between 48 and 88, mean postmortem delay was 7 hours, and mean disease duration was 29.3 years. Adult control and MS astrocytes were purified from post-mortem subcortical wm of non-demented healthy subjects and MS patients and cultured as described^15,39^.

#### Organotypic cerebellar slice cultures

Neonatal meninges-free rat cerebella of either sex were cut in 300 μm sagittal sections and cultured in millicell cell culture inserts as described^22,55^. After 3 weeks, slices were left untreated or exposed to lysolecithin (0.5 mg/mL) for 17 hours to induce demyelination (day 0). At 2 days post lysolecithin (DPL) treatment, control and demyelinated slices were left untreated or exposed to Poly(I:C) (50 μg/mL) for 48 hours. At 5 DPL, the tissue slices were either fixed in 4% paraformaldehyde (PFA) or homogenized in TE‐buffer (10 mM Tris, 2 mM EDTA, pH 7.4) containing 0.25 M sucrose and a mix of protease inhibitors (Complete Mini) on ice, or left to examine remyelination until 21 DPL.

### Biochemical analysis

#### Western blotting

Cells were lysed in TNE-lysis buffer containing 150 mM NaCl, 50 mM Tris-HCl and 5 mM EDTA (pH 7.4) supplemented with 1% Triton X-100, and protease inhibitors. Extracellular deposits were obtained by lysing the cells in water for 2 hours at 37 °C and scraping the remaining deposits in deoxycholate (DOC)-containing buffer (2% DOC, 2 mM EDTA, 5 mM Tris, pH 8.0). Following 30 minutes of incubation in lysis buffer (cells) or DOC-buffer (deposits) on ice, protein concentrations were determined by a BioRad DC-protein assay using bovine serum albumin (BSA) as a standard. To separate DOC-soluble (fibronectin dimers) and -insoluble (fibronectin aggregates) proteins, equal amounts of protein of DOC-extracts were centrifuged at 16,000 g for 30 minutes at 16 °C. Samples were subjected to SDS-PAGE under reducing (cells, 20 µg) or non-reducing conditions (deposits, 12 µg) to detect total fibronectin monomers or structural states of fibronectin, including aggregates and dimers, respectively. After transfer of the proteins to a PVDF membrane (Immobilon-FL) and blocking with 50% Odyssey blocking buffer in phosphate buffered saline (PBS) the membranes were incubated overnight at 4 °C with the indicated primary antibodies (Suppl. Table S1). Appropriate secondary IRDye-conjugated antibodies were applied for 1 hour at room temperature followed by detection on the Odyssey Infrared Imaging system. Membrane washing between antibody incubations was performed with PBS supplemented with 0.05% Tween-20. Quantification was performed with FUJI ImageJ software.

#### Immunoprecipitation

Astrocytes were washed with ice-cold PBS and incubated in ice-cold sulfo-NHS-LC-biotin (0.1 mg/mL) for 1 hour on ice to biotinylate cell surface proteins. After three washes in cell wash buffer (65 mM Tris, 150 mM NaCl, 1 mM CaCl_2_, 1 mM MgCl_2_, pH 7.5), and two washes in PBS, the cells were scraped in TNE lysis buffer followed by sonication. Lysates (100 µg) were precleared by incubating lysates with rabbit Ig (rabbit gamma globulin 11.1 mg/mL), or mouse Ig (mouse gamma globulin 11.0 mg/mL), and 20 µl Protein A/G Plus-agarose beads for 1 hour at 4 °C. Precleared lysates were incubated overnight with anti-integrin antibodies (Suppl. Table S1) at 4 °C. Hamster-anti-β1 integrin was first linked to rabbit-anti-hamster for 30 minutes at room temperature. The samples were incubated with Protein A/G Plus-agarose beads for 3 hours at 4 °C. The beads were precipitated for 5 minutes, at 600 g, washed four times with immunoprecipitation wash buffer (cell wash buffer supplemented with 350 mM NaCl and 1% NP-40) and resuspended in non-reducing sample buffer. The samples were heated to 95 °C for 3 minutes, centrifuged for 5 minutes at 600 g, followed by Western blotting and detection of surface integrins using IR-dye-800-conjugated streptavidin.

### Adhesion assay

Adhesion assays were performed as described^78^. For integrin blocking experiments, cells were incubated with the indicated anti-integrin antibodies for 30 minutes, at 37 °C. For cytokine pre-incubation experiments, cells were left untreated or pre-treated for 1 hour with cytokine mixture at 37 °C, followed by plating and stimulation with Poly(I:C) and/or MnCl_2_ (1 mM). Astrocytes were seeded at a density of 50,000 cells per well and left to adhere for 2 hours at 37 °C. Adhesion is expressed as percentage of the corresponding untreated cells or relative to MnCl_2_-treated cells.

### Immunochemistry

#### Immunohistochemistry

Cerebellar slices were fixed for 1 hour in 4% PFA. After two washes with PBS the slices were blocked for 3 hours in Hanks Balanced Salt Solution supplemented with 1 mM HEPES, 10% heat inactivated normal goat serum, 2% heat inactivated horse serum, 1% BSA and 0.25% Triton X-100. Slices were incubated with the indicated primary antibodies in blocking solution for 48 hours at 4 °C (Table S1), washed three times in PBS supplemented with 0.05% Tween for 1 hour and incubated with appropriate Alexa Fluor© secondary antibodies (1:500) overnight at 4 °C. After three washes the slices were mounted on glass slides using mounting medium to prevent image fading. Imaging were performed on a Leica TCS SP2 AOBS confocal microscope. The percentage of myelinated axons was calculated using MATLAB software as described^22^.

#### Immunocytochemistry

For live cell stainings, cells were blocked for 10 minutes with 4% BSA and incubated with anti-fibronectin antibodies (Suppl. Table S1) for 30 minutes on ice. After three washes in PBS, cells were incubated with the appropriate Alexa Fluor© secondary antibodies for 20 minutes on ice. Cells were fixed with 4% PFA and incubated with DAPI (1 μg/mL) for 15 minutes at room temperature. For staining of fixed cells, cells were first fixed sequentially with 2% and 4% PFA for 15 minutes each, and subjected to the indicated primary and secondary antibodies as described for live staining. Analysis and imaging were performed on a conventional Leica DMI 6000 B immunofluorescence microscope. Astrocytes were scored based on their cellular surface extracellular fibronectin expression and subdivided into two categories, i.e., diffuse fibronectin staining in small, punctuated structures and fibronectin in large, elongated structures. Between 100–175 cells were counted per condition.

### Polymerase chain reaction

Cells were scraped in RNA protect (Qiagen) and mRNA was isolated using an mRNA-isolation kit (Isolate II RNA Micro Kit; Bioline) according to manufacturer’s instructions. Total RNA (1 μg) was reverse transcribed in the presence of oligo(dT)12–18 and dNTPs with M-MLV reverse transcriptase according to manufacturer’s instructions. Primer sequences are shown in Supplementary Table S2.

#### RT-PCR

cDNA was amplified using indicated primer sets and GoTaq Green Master Mix (Promega) according to manufacturer’s instructions, and subjected to a 1.5% agarose gel. The EIIIA- and EIIIB-fibronectin primers generate two products: a band that includes the alternatively spliced domain (526 bp for *EIIIA*^*pos*^*-Fn*, 640 bp for *EIIIB*^*pos*^*-Fn*) and another band when the respective domain is spliced out (256 bp for *EIIIA*^*neg*^*-Fn*, 367 bp for *EIIIB*^*neg*^*-Fn*^79,80^). Quantification was performed with FUJI ImageJ software. Relative expression of *EIIIA*^*pos*^- and *EIIIB*^*pos*^-*Fn* is expressed as percentage of total *Fn* (positive and negative bands combined).

#### qPCR

Gene expression levels were measured by real-time quantitative RT-PCR using ABsolute QPCR SYBR Green Master Mix in a Step-One Plus Real-Time PCR machine. Measurements were performed in triplicate and amplification data was processed using LinRegPCR software^81^.

### Statistics

Data are expressed as mean ± standard error of the mean (SEM) of at least three independent experiments. A Shapiro-Wilk normality test was first applied to test the normal distribution of the data. When normality failed, a Wilcoxon Signed Rank test or Mann Whitney test was used to test for statistical significance where indicated. When normality passed, statistical analysis was performed with a one-sample t-test when relative values of groups were compared with control by setting the untreated control values at 1 in each independent experiment. When values between two treatment groups were compared, statistical significance was assessed using a paired two-sided t-test or a one way ANOVA with a Šidák multiple comparisons post-test when comparing more than two groups. When gm and wm astrocytes were compared, wm astrocyte control values were set at 1. Here, statistical significance compared to wm control was performed using a one sample t-test (*), and a one way ANOVA with a Dunnett post-test was used to compare between gm samples and gm control (#). In case of human astrocyte cultures, arbitrary measured values were normalized to control values, with an arbitrary average control value of 1. Statistics were performed using GraphPad Prism 6.0. In all cases p-values of <0.05, <0.01, and <0.001 were considered significant and indicated with *, **, *** or #, ##, ###, respectively.

## Supplementary information

Supplementary information.

## Data Availability

All data generated during and/or analysed during the current study are available from the corresponding author on reasonable request.
