# Supplementary material for: TLR3 agonists induce fibronectin aggregation by activated astrocytes: a role of pro-inflammatory cytokines and fibronectin splice variants
Source: Sci Rep. 2020 Jan 17;10:532. doi: 10.1038/s41598-019-57069-4 (PMC6969115; doi:10.1038/s41598-019-57069-4)
Supplement: Supplementary file 1 — Supplementary information. [file 41598_2019_57069_MOESM1_ESM.pdf]

Supplementary information

# TLR3 agonists induce fibronectin aggregation by activated astrocytes: a role of pro-inflammatory cytokines and fibronectin splice variants

*Inge Werkman[1,‡], Arend H. Sikkema[1,‡], Joris B. Versluijs[1], Jing Qin[1], Pascal de Boer[1], and Wia Baron[1,*]*

[1] Department of Biomedical Sciences of Cells & Systems, section Molecular Neurobiology, University of Groningen, University Medical Center Groningen, Groningen, The Netherlands

[‡] These authors contributed equally to this work

**Contents**

| | Page |
|---|---|
| **Supplementary Tables** | |
| Table S1 - primary antibodies used | 2 |
| Table S2 - primer sequences used | 3 |
| **Supplementary Figure** | |
| Figure S1 | 4 |
| Figure S2 – full scans of immunoblots | 5 |
| Figure S3 – full scans of immunoblots | 6 |
| Figure S4 – full scans of agarose gels | 7 |

**Table S1**. Primary antibodies used during western blotting, immunoprecipitation, immunohistochemistry and immunocytochemistry

| protein | dilution | catalog # | manufacturer |
|---|---|---|---|
| actin | 1:1000 | A5441 | Sigma |
| $EIIIA^{pos}/EDA^{pos}$-fibronectin | 1:500 | IIIE2/F6140 | Sigma |
| $EIIIB^{neg}$-fibronectin | 5 µg/ml | IST6/S-FN9 | Sirius Biotech |
| fibronectin | 1:100/1:500 | AB2033 | Millipore |
| GFAP | 1:5000 | Z033430 | DAKO |
| iNOS | 1:250 | 610329 | BD biosciences |
| MBP (immunohistochemistry) | 1:250 | MCA409S | Bio-Rad |
| MBP (western blot) | 1:200 | MAB386 | Millipore |
| neurofilament | 1:5000 | CPCA-NF-H | Encor Biotechnology Inc. |
| TuJ1 | 1:1000 | kind gift of Dr. A. Frankfurter | University of Virginia |
| β1 integrin | 1:125 | 555003 | BD Biosciences, |
| β3 integrin | 1:125 | 554951 | BD Biosciences |
| β5 integrin | 1:125 | MAB1961 | Millipore |

**Table S2.** Primer sequences used for RT-PCR and qPCR

| gene | species | forward primer | reverse primer | product size (bp) |
|---|---|---|---|---|
| *EIIIA -Fn* | rat | 5’-AAACAGAAATGACCATTGAAGGTTTG-3’ | 5’-TTGATTTCTTTCATTGGTCCTGTCTT-3’ | pos 526 neg 256 |
| *EIIIB -Fn* | rat | 5’-TTACACTGTCAAAGATGACAAGGAAA-3’ | 5’-TGACATCAGAAGAATCAAAACCAGTT-3’ | pos 641 neg 368 |
| *EDA-FN* | human | 5’-AGTAACCAACATTGATCGCCC-3’ | 5’-CCTGTACCTGGAAACTTGC-3’ | 106 |
| *EDB-FN* | human | 5’-ACAACAAACGGCTGTTCCTC-3’ | 5’-CCAGGAAGTTGGTTAAATCAATGG-3’ | 113 |
| *FN1* | human | 5’-GATAAATCAACAGTGGGAGCGG-3’ | 5’-GTCTCTTCAGCTTCAGGTTTACTC-3’ | 108 |

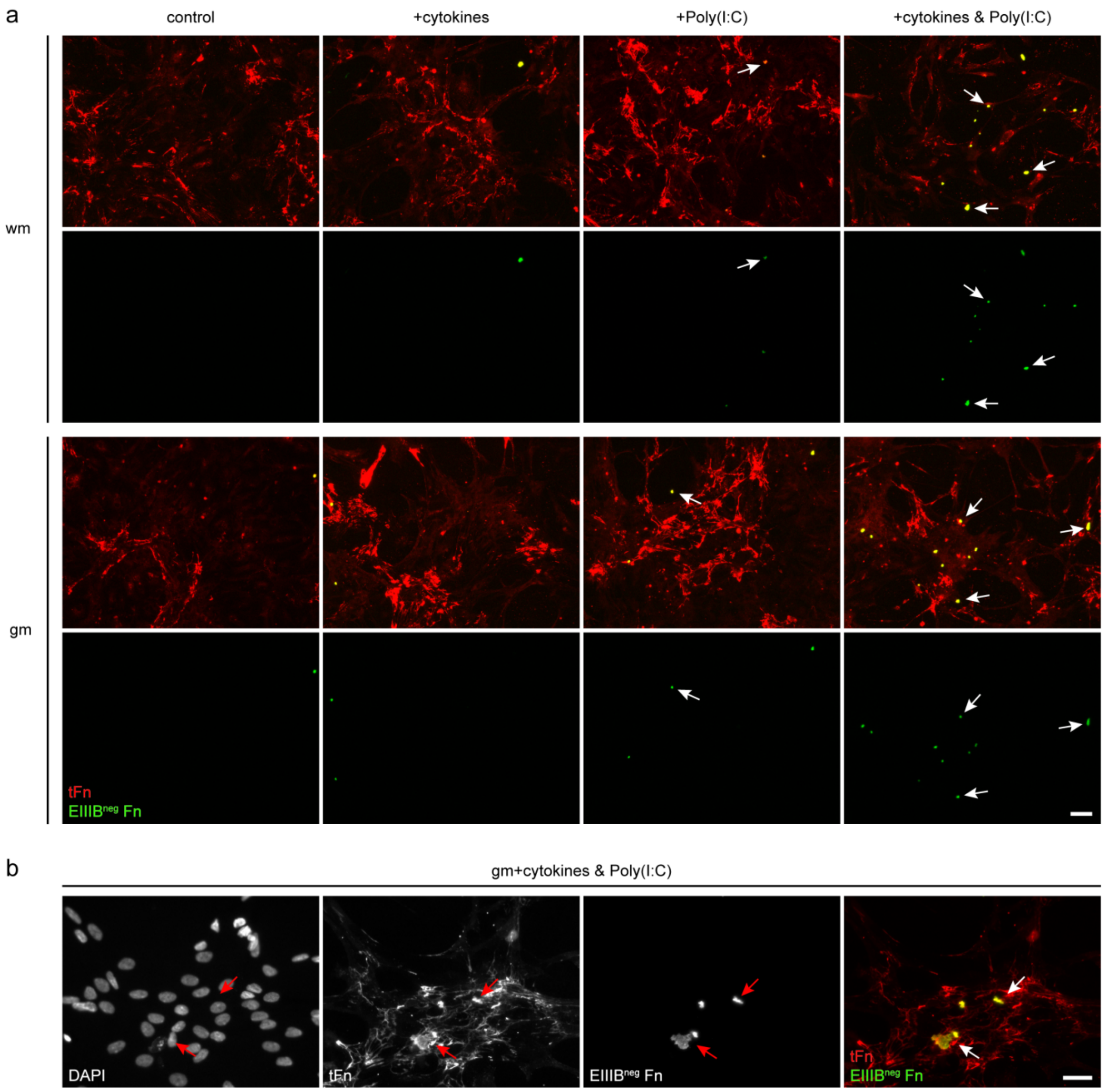


**Fig. S1** Fibronectin aggregates contain EIIIB$^{neg}$-fibronectin. **a,b** Immunocytochemistry of fixed, unpermeabilized grey matter (gm) and white matter (wm) rat astrocytes. Cells were pre-incubated for 24 hours with a mixture of IFNγ (500 units/mL), IL1β (10 ng/mL) and TNFα (10 ng/mL), followed by Poly(I:C) (50 μg/mL) treatment for 48 hours. Cells were stained for total fibronectin (tFn, red) and EIIIB$^{neg}$-fibronectin (EIIIB$^{neg}$-Fn, green). Note the extracellular EIIIB$^{neg}$-fibronectin-containing structures in between cells that were pre-incubated with cytokines and treated with Poly(I:C) (arrows), while EIIIB$^{neg}$-fibronectin is hardly observed at the cells surface. Scale bar is 75 μm (**a**), or 25 μm (**b**).

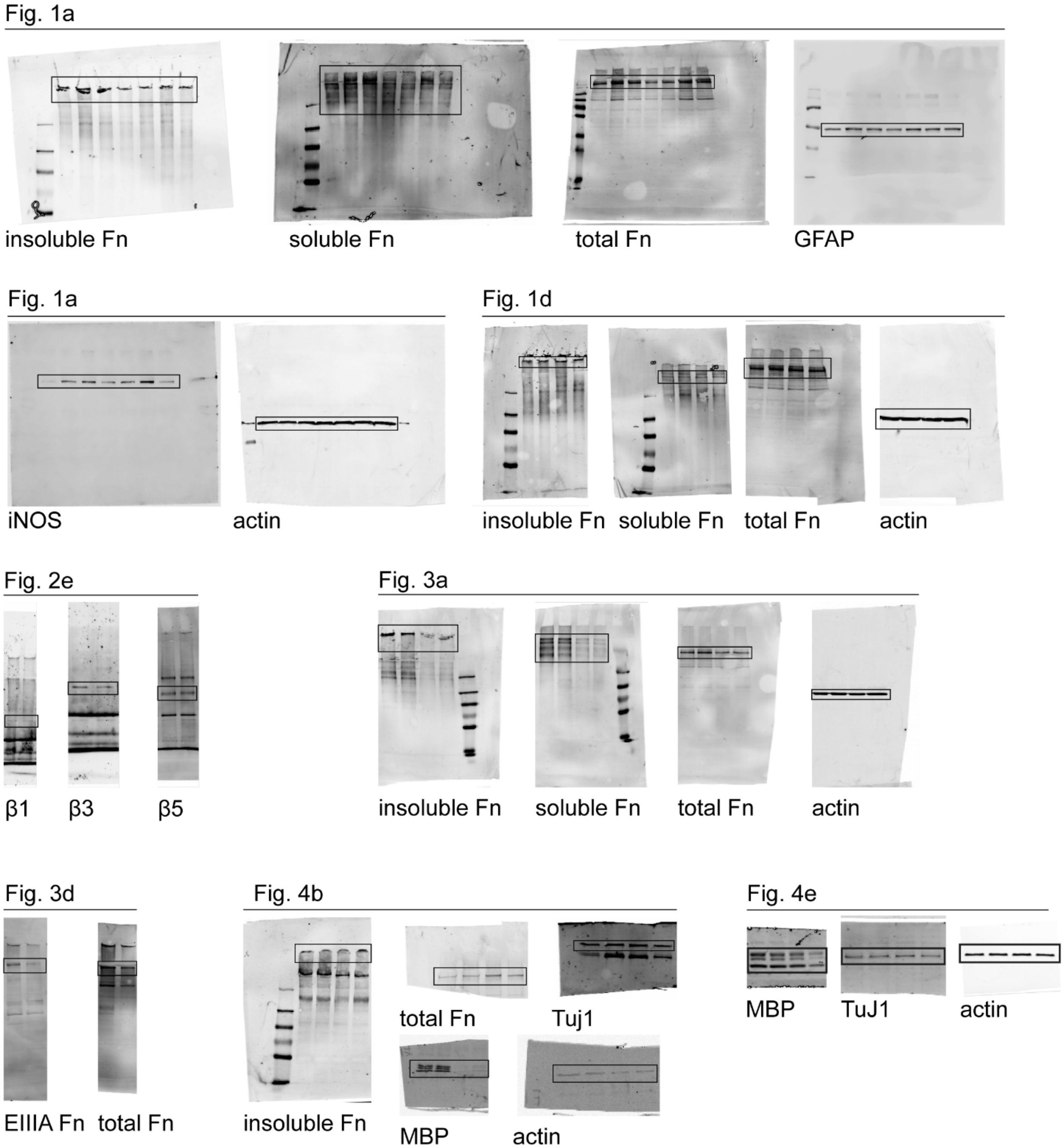


**Fig. S2** Full scans of indicated immunoblots. The boxed areas are presented in the indicated figures.

Fig. 5b

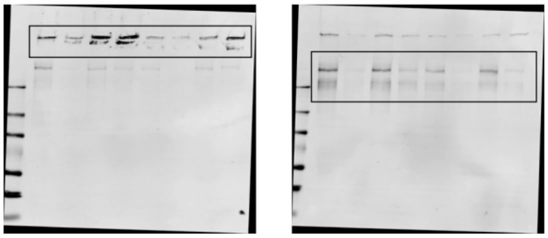

insoluble Fn soluble Fn

Fig. 6b

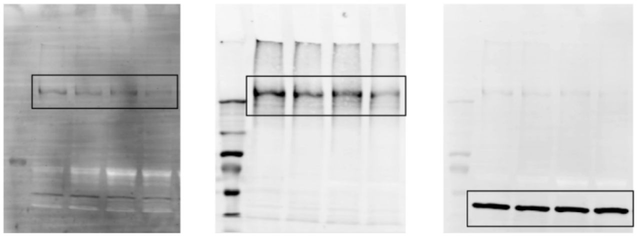

EIIIA total Fn actin

Fig. 7a

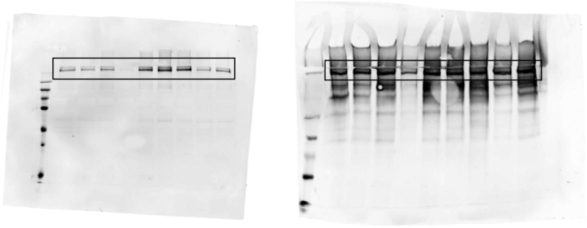

EIIIA total Fn

**Fig. S3** Full scans of indicated immunoblots. The boxed areas are presented in the indicated figures.

Fig. 6e

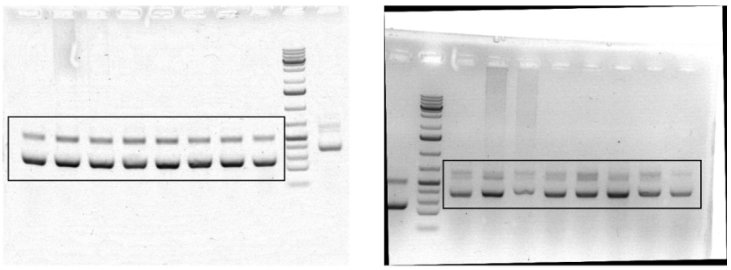

EIIIA EIIIB

**Fig. S4** Full scans of indicated agarose gels. The boxed areas are presented in the indicated figures.
